# Graves' disease with only unilateral involvement; a case report

**DOI:** 10.1016/j.ijscr.2023.109138

**Published:** 2023-12-09

**Authors:** Mohammed Saad Bu Bshait

**Affiliations:** Department of Surgery, College of Medicine, King Faisal University, Saudi Arabia

**Keywords:** Hyperthyroidism, Unilateral Graves' disease, Case report

## Abstract

**Introduction:**

Graves' disease characteristically presents with a diffuse goiter secondary to the autoantibodies that target the thyrotropin receptors of the thyroid gland. Few cases have been reported of only one of the two lobes being affected. The cause of this phenomenon is still uncertain. Here we report on another case of unilateral Graves' disease.

**Case presentation:**

A 43-year-old female patient presented with a history of weight loss, palpitations and right sided neck swelling for 4 months. Clinical examination showed an enlarged right thyroid lobe. Laboratory investigations yielded evidence of thyrotoxicosis with suppressed thyroid stimulating hormone. In addition, anti-TSH receptor and anti-thyroperoxidase antibodies were positive. Neck Ultrasound showed an enlarged right thyroid lobe with increased vascularization. The isthmus and left lobe were both normal in size. A Tc^99m^ pertechnetate thyroid scan demonstrated enlargement of the right thyroid lobe with diffuse intense uptake, whereas the left lobe was suppressed. A diagnosis of unilateral Graves' disease was made. The thyrotoxicosis was treated and maintained with methimazole.

**Discussion:**

Unilateral Graves' disease is a rare manifestation of Graves' disease, sharing the same autoimmune background and the symptoms of thyrotoxicosis. Enlargement of only one lobe was evident on clinical examination. The distinctive feature was unilateral uptake during thyroid scintigraphy. The exact pathophysiology of this condition has yet to be elucidated. Management options and responses are similar to those of classical Graves' disease.

**Conclusion:**

Unilateral uptake during thyroid scintigraphy and/or unilateral lobar goiter in the setting of hyperthyroidism can be the presentation of unilateral Graves' disease.

## Highlights

• Unilateral involvement is a rare presentation of Graves' disease.
• The cause is still uncertain.
• Variable lobar reaction to TSH stimulation is a possibility.
• Resistance of TSH receptors to TSH autoantibodies is another speculation.
• Management options are like that of classical Graves' disease.

## 1 Introduction

Graves' disease is an autoimmune condition in which the thyrotropin receptor is stimulated by circulating autoantibodies, leading to excessive release of thyroid hormones. The homogeneous exposure of the gland to these circulating antibodies characteristically affects it in a diffuse manner. This broad involvement is evident as diffuse goiter, with sonographic hypervascularity, and universally intense uptake on scintigraphy [1]. In contrast, Graves' disease with unilateral involvement in a bilobed gland has been reported only occasionally. Here we describe another rare case of unilateral Graves' disease. This work has been reported in accordance with the SCARE criteria [2].

## 2 Case presentation

#### 2.1.1 Patient information

A 43-year-old female patient presented with a history of weight loss despite an increase in appetite, palpitations and a right sided neck swelling of four months' duration. The patient denied compression symptoms or a family history of thyroid cancer.

#### 2.1.2 Clinical findings

There was enlargement of the right thyroid that moved with deglutition. There was no retrosternal extension nor lymph node enlargement. The patient had no exophthalmos or pretibial myxedema.

## 3 Diagnostic assessment

#### 3.1.1 # Thyroid function test

T3 and T4 were elevated with suppressed TSH. T3 was 17.1 pmol/L (Reference range: 3–6.9), T4 was 37.5 pmol/L (Reference range: 12–22), and TSH was <0.005 uIU/ml (Reference range: 0.35–4.9).

Anti-TSH receptor (TRAb) and anti-thyroperoxidase (TPO-Ab) antibodies were positive.

#### 3.1.2 # Neck ultrasonography

**Fig. 1:**
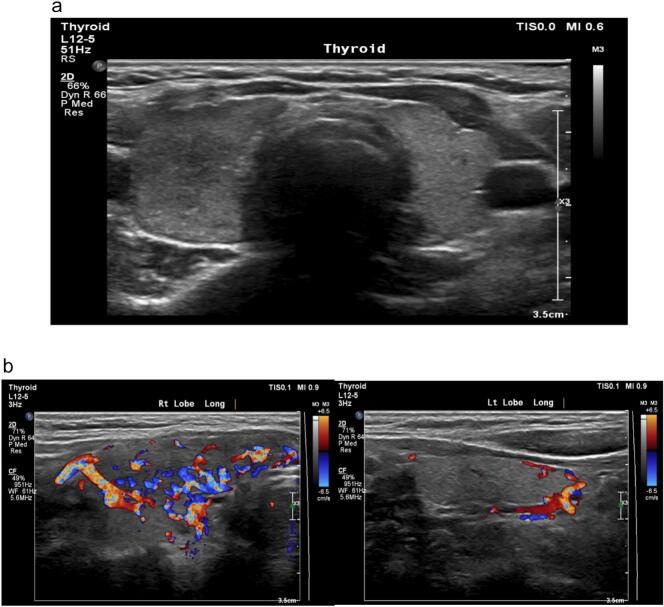
a. Neck ultrasound showing enlarged right thyroid lobe. b. Color doppler neck ultrasound demonstrating the greater hypervascularity of right thyroid lobe.

This showed an enlarged right thyroid lobe with a normal isthmus and left lobe. Both lobes were hypervascular but this was more evident on the right. (Fig. 1a–b).

#### 3.1.3 # Thyroid scintigraphy

**Fig. 2:**
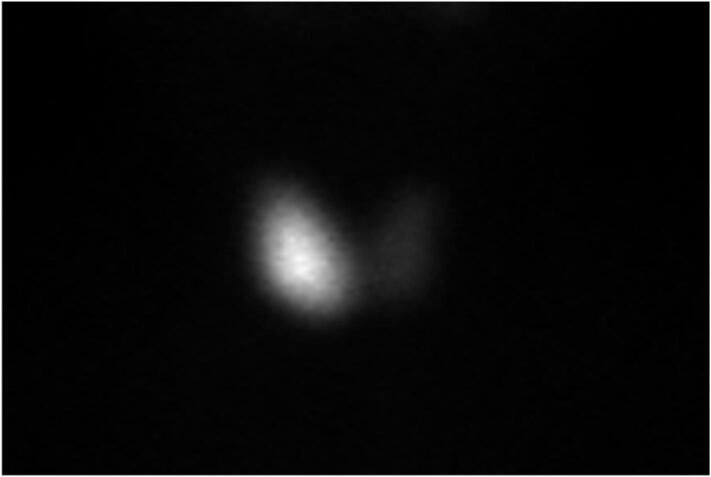
Tc^99m^ pertechnetate thyroid scan demonstrated enlarged right thyroid lobe with diffuse homogeneous dense uptake. Left thyroid expressed radiotracer suppression. Total thyroid uptake was 8.4 %. Right and left lobe uptake were 7.1 % and 1.3 % respectively.

A Tc^99m^ pertechnetate thyroid scan showed diffuse hyperemia and enlargement of the right thyroid lobe. Uptake was homogeneously dense with a smooth outline. In contrast, the left thyroid lobe was normal in size with homogeneous radiotracer suppression. Total thyroid uptake was 8.4 %. Right and left lobe uptake was 7.1 % and 1.3 % respectively. (Fig. 2).

### 3.2 Therapeutic intervention

The patient was started on antithyroid medication (Methimazole) to control thyrotoxicosis.

### 3.3 Follow-up and outcome

Thyrotoxicosis regressed and patient stayed in normal status for the next four months. Based on the patient preference, she was referred to surgery clinic for considering the option of surgical management. However, surgical intervention has not yet been carried out due to patient hesitancy.

## 4 Discussion

Unilateral involvement of thyroid lobes is a rare presentation of Graves' disease. Skata and colleagues reported the first two cases of unilateral Graves' disease [3]. The first case presented with right nodular goiter and euthyroid status but with positive antimicrosomal antibodies. Thyroid ultrasound revealed an enlarged right thyroid lobe with a hypoechoic nodule. Thyroid scintigraphy with Tc^99m^ showed intensive uptake in the right lobe. The patient underwent right hemithyroidectomy on suspicion of malignancy. Surprisingly, histopathological evaluation was concordant with Graves' disease. Subsequently, the patient presented after 27 months with symptoms of thyrotoxicosis, an enlarged contralateral lobe, elevated thyroid function tests, low TSH and positive TRAb which had been negative preoperatively. A thyroid scintigram with ^123^I-uptake showed an increase in uptake in the remaining left thyroid lobe. The second case was a 61-year-old female patient who presented with weight loss, palpitations and elevated thyroid function tests but negative TRAb. Ultrasound excluded the presence of nodules, with a normal gland size. Thyroid ^123^I-uptake showed diffuse unilateral uptake in the right lobe. Given the possibility of a diffuse hot nodule, the patient underwent right hemithyroidectomy. Histopathology was compatible with Graves' disease. Eight months following surgery, thyrotoxic symptoms developed, with laboratory findings and increased thyroid scintigraphy uptake in the left lobe comparable with the first case.

Since then, few other cases have been reported [[4], [5], [6], [7], [8]]. In these, clinical symptoms of thyrotoxicosis are similar to those in ordinary Graves' disease, including ophthalmopathy. It might be different in selective lobe enlargement which may mimic toxic adenoma or Plummer's disease. This distinctive presentation was seen in the current case as well as others [[3], [4], [5], [6], [7], [8]]. On the other hand, one reported case showed no difference in the size of both lobes [3]. All reported cases demonstrated high thyroid function tests with low TSH, except for one case of Saka et al. in whom thyroid function tests and TSH were initially normal. Thyroid autoantibodies displayed disparate results. This is consistent with what is found in classical Graves' disease. In the current patient, both TPO-Ab and TRAb were positive in a manner comparable to several other cases of unilateral Graves' disease [[4], [5], [6]]. Cytological evaluation was carried out in two of the reported cases, showing lymphocytic infiltration of the affected right lobe but not the left [4,5]. Unilateral uptake on scintigraphy was the distinctive feature of unilateral Graves' disease. All except one [7] involved the right lobe in a manner identical to our patient. A larger right thyroid lobe in a normal gland, and a preponderance of left lobe involvement in thyroid hemiagenesis, suggests the possibility of predominant right lobe organogenesis and functional advantages over the left [5]. Furthermore, a study evaluating lobar activity in Graves' disease with diffuse intense TC^99m^ uptake showed that the right thyroid lobe expressed a higher uptake than the left [9].

The cause of unilateral Graves' disease is not yet known. Various possibilities have been suggested. Since TSH stimulation affects thyroid gland function and growth, a variable lobar reaction may be present in unilateral Graves' disease [10]. This functional and/or structural variation might be caused by a pre-existing congenital condition (such as bilobed isolated lymphatic drainage) or by acquired conditions (such as previous bacterial or viral thyroid inflammation) [11]. Impaired thyroidal radioiodine uptake as a result of local suppression of sodium iodide symporter gene expression is another speculation [12]. Lobar differences in sensitivity to TSH stimulation is another possibility. Muller-Gartner and colleagues evaluated the resistance of TSH receptors to TSH autoantibodies in 256 patients with Graves' disease. They found single or several foci of resistance to TSH-receptor autoantibodies in about 5 % [13]. The relationship of the aforementioned ideas to the development of unilateral Graves' disease is uncertain and further investigation is needed.

In spite unilateral lobar involvement, management should be guided in the same way as with ordinary Graves' disease. As in the current patient, most of the cases reported in the literature were treated and responded well to antithyroid medication [[4], [5], [6], [7]]. Bolognesi and Rossi [6] reported the findings of a Tc^99m^ scan carried out after two years of medical treatment. This showed almost identical scanning pattern of the affected right lobe but a trace of weak uptake in the left lobe which had initially been suppressed. Chen et al. [8] had managed their patient with radioiodine ablation. Following ablation, ^131^I uptake revealed a homogeneous normal uptake in the initially suppressed right thyroid lobe with small amount seen in the affected left lobe. Hemithyroidectomy was performed by Saka et al. for reasons other than Graves' disease. The remaining lobe was diseased, as shown by clinical, laboratory and thyroid scintigraphy findings. Nevertheless, disease regression was successfully achieved with antithyroid medication. Thus, when surgical management is selected for unilateral Graves' disease, the optimal surgical intervention and possibility of recurrence should be taken into consideration.

## 5 Conclusion

Unilateral uptake on thyroid scintigraphy and/or selective lobar goiter in the context of hyperthyroidism could be the presentation of unilateral Graves' disease. Collection of clinical, laboratory and imaging results are needed to distinguish this rare manifestation of the disease. Management procedures are identical to those adopted in the classical form of Graves' disease including the extent of surgery.

## Consent

Written consent was obtained from the patient for publication of this case report.

## Ethical approval

Approval has been granted by the Research Ethics Committee at King Faisal University, Alahsa, Saudi Arabia. (Reference No. KFU-REC-2023-OCT-ETHICS1626 on 13 October 2023).

## Funding

There are no sponsors or special funding for writing or publication of this case report.

## Author contribution

Not applicable.

## Guarantor

Mohammed Saad Bu Bshait.

## Declaration of competing interest

Conflict of interest is denied.
